# Prevalence of lectures about dental esthetics and female speakers in three Brazilian conferences

**DOI:** 10.1590/0103-6440202305349

**Published:** 2023-07-17

**Authors:** Karen Larissa Oliveira Conceição, Karla Danielly Alves Soares, Rafaella Mariana de Bragança, Marcos Britto Correa, Rafael Ratto Moraes, André Luis Faria-e-Silva

**Affiliations:** 1 Department of Dentistry, Universidade Federal de Sergipe, Aracaju, SE, Brazil; 2 Graduate Program in Dentistry, Universidade Federal de Pelotas, Pelotas, RS, Brazil; 3 Graduate Program in Dentistry, Universidade Federal de Sergipe, Aracaju, SE, Brazil

**Keywords:** Dental education, Esthetics, Gender differences, Oral diseases

## Abstract

This study analyzed the prevalence of lectures involving esthetics in the scientific program of Brazilian dental conferences and the gender distribution of speakers. All lectures presented in three dental conferences (Bahia, São Paulo, and Goiás states) held from 2016 to 2020 were evaluated. Three investigators individually divided the lectures according to the specialties recognized by the Brazilian Federal Council of Dentistry (FCD) based on their titles. The lectures were also classified as involving or not esthetics, and the speaker`s gender was recorded. Descriptive statistical analyses were performed, and Chi-square tests assessed possible associations between factors. The words most cited in the titles of the lectures were “esthetic” (13.6%), “dentistry” (9.9%), and “treatment” (8.1%). Oral diseases were barely mentioned in the titles (up to 1.3%). The highest number of lectures was observed for the specialty of Restorative Dentistry (22.3%), followed by Prosthodontics (18.5%). Approximately one-third of lectures involved some aesthetic aspect, but this percentage ranged from 71.9 to 78.6% for the two specialties with more lectures. Regarding the speaker`s gender, the inequity was higher for lectures involving esthetics (81.6% of males) than for topics unrelated to esthetics (66.7%). More male speakers than females were observed for all specialties. The highest gender gap was observed for Pediatric Dentistry with 62.4% male speakers, although only 10.6% of FCD registered specialists were men. In conclusion, the Brazilian dental conferences analyzed seemed to favor offering lectures dealing with esthetic topics and male speakers.

## Resumo

Este estudo analisou a prevalência de palestras envolvendo estética na programação científica de congressos de Odontologia brasileiros, e a distribuição do gênero dos palestrantes. Todas as palestras apresentadas em três congressos de Odontologia (CIOBA, CIOGO, and CIOSP) entre 2016 e 2020 foram avaliadas. Três avaliadores utilizaram os títulos das palestras para dividi-las entre as especialidades reconhecidas pelo Conselho Federal de Odontologia (CFO) do Brasil. As palestras foram também classificadas como envolvendo ou não estética, e o gênero do palestrante foi registrado. Análises estatísticas descritivas foram realizadas, e testes de Qui-quadrado avaliaram possíveis interações entre fatores. A palavra mais citada nos títulos das palestras foi “estética” (13,6%), seguido por “odontologia” (9,9%) e “tratamento” (8,1%). Doenças orais foram raramente mencionadas nos títulos (até 1,3%). O maior número de palestras foi observado para a especialidade de Dentística (22,3%), seguido por Prótese Dental (18,3%). Aproximadamente um terço das palestras envolviam algum aspecto estético, com porcentagens entre 71,9 a 78,6% para as duas especialidades com mais palestras. Em relação ao gênero do palestrante, a inequidade foi maior para palestras envolvendo estética (81,6% de homens) que para tópicos não relacionadas à estética (66,7%). Mais palestrantes do sexo masculino que feminino foram observados para todas as especialidades. A maior discrepância entre os gêneros foi observada para Odontopediatria, uma especialidade com 62,4% dos palestrantes do sexo masculino, embora apenas 10,6% dos especialistas registrados no CFO eram homens. Como conclusão, os congressos de Odontologia Brasileiros parecem preferir palestras abordando tópicos de estética e palestrantes do sexo masculino.

## Introduction

Brazil has more than 380,000 dentists registered in its Federal Council of Dentistry (FCD), representing approximately a fifth of the world`s dentists ^1^. This high number of dentists resulted in a 570 inhabitants/dentist ratio, which is almost three-fold that recommended by the World Health Organization (WHO) as sufficient to provide adequate health care to a population ^2^. Despite the excess of dentists, an unplanned expansion of dentistry courses in Brazil has been observed in the last decades ^3^. In 2020, there were 544 authorized courses in the country, which are higher than those observed in more populated countries such as India (313), China (96), and the United States of America (67) ^4^ ^,^ ^5^ ^,^ ^6^. Despite the high number of dentists, Brazil presents approximately 45% of its population with some oral disorder ^7^, which is a higher rate than that observed for the worldwide population (40%) ^8^. Indeed, dentists' distribution in the country is not based on the oral health needs of the population ^9^, and most clinicians prefer to work in more profitable private clinics in large cities ^10^. In this scenario, an enhanced number of overtreatments and the seeking for new modalities of interventions for dentists can be expected in a very competitive labor market, as observed in Brazil.

Despite this population health problem, as observed in other primarily health-based professions, the number of merely aesthetic procedures has increased worldwide ^11^ ^,^ ^12^ ^,^ ^13^. Emotional, psychological, and practical (e.g., reduce time spent with makeup) motivations were some common reasons reported by patients seeking esthetic procedures ^14^. An improved attractiveness achieved with esthetic procedures can positively affect the patient's quality of life, social interactions, and perspective of obtaining higher-paying jobs ^15^ ^,^ ^16^. Social media can also contribute to the surge in patients seeking esthetic treatments in dentistry. A prior study showed that most general practitioners believe that social media is important for communicating with patients, and approximately half of the clinicians use the platforms for advertising content related to esthetic dentistry ^13^. Following this tendency, clinical procedures to obtain facial proportions, lip balance, chin-nose balance, and others started to be performed by dentists as routine. In Brazil, the FCD introduced Orofacial Harmonization as a new dental specialty in 2019. Since new esthetic techniques and materials are frequently developed, clinicians must attend several continuing education courses to apply these novelties in their practice.

The patient`s gender has been reported as an important factor in seeking esthetic procedures, and women tend to desire esthetic improvements more than men ^14^ ^,^ ^17^ ^,^ ^18^. Regarding gender, most dentists registered in the UK (56%) and Australia (59%) are males. Still, a larger discrepancy is observed when the male-to-female speaker ratio in dental conferences is assessed in these countries ^19^ ^,^ ^20^. Conversely, most dentists are females in Brazil (only 43% are men) ^1^, and a lower gender gap among the speakers at dental conferences would be expected. Prior studies have shown gender inequalities in scientific publications by Brazilian dental researchers ^21^ ^,^ ^22^, but information about the gender gap among speakers at Brazilian dental conferences is currently unavailable. The present study assessed the prevalence of lectures involving esthetic topics and gender speakers in three major dental conferences held in Brazil between 2016 and 2020.

## Materials and methods

### Experimental design

This is a cross-sectional study using 5-year retrospective data from three selected major Brazilian dental conferences (convenience sampling). We selected three dental conferences held in different geographic Brazilian regions: Northeast, Southeast, and Central-West. Then, the International Dental Conferences held in the states of São Paulo (CIOSP), Bahia (CIOBA), and Goiás (CIOGO) were selected. All official scientific programs of conferences from 2016 to 2020 were evaluated. Lectures were classified according to the 23 dental specialties defined by the FCD and the topic (involving or not any aesthetic aspect). The gender of the speaker was also recorded. The outcomes were the relative and absolute prevalence of lectures/ speaker genders per specialty and esthetic involvement.

### Eligibility criteria

Major dental conferences attended by a large number of dentists, not limited to specific dental specialties or topics, were selected. No more than one conference by Brazilian geographic region was selected, and all lectures described in the official scientific programs between 2016 and 2020 were assessed. Unlike CIOSP (annual conference), the other conferences occur every two years. Then, the scientific programs of CIOGO held in 2017 and 2019 were assessed, while the years evaluated for CIOBA were 2016 and 2018. The CIOBA 2020 did not take place due to the COVID-19 pandemic.

### Preliminary evaluators training

The official scientific programs were obtained through the respective websites or required by e-mail. Three evaluators were previously trained, seeking to reduce discrepancies and misclassifications. For this purpose, the evaluators individually classified at least a hundred random lectures regarding their dental specialty and involvement of esthetics. A list of keywords for each specialty and classification of esthetic involvement was defined based on the results of this preliminary training. For instance, the presence of “children”, “deciduous”, “Pulpotomy”, or “tooth eruption” in the title indicated that the lecture was related to “pediatric dentistry”.

### Lectures classification by dental specialty

In the first step of the data extraction, the three-trained evaluators individually divided the lectures into the 23 dental specialties defined by the FCD of Brazil. The classification was based only on the lecture`s title, and the same lecture could be allocated for more than one dental specialty. Broad topics involving several dental specialties and those not restricted to dentistry (e.g., Marketing) were classified as “other”. Discrepancies were solved by consensus after discussion, and the criteria adopted for the classification were followed during the entire study.

### Lectures classification by esthetic involvement

 Afterward, the lectures were classified as involving or not esthetic. Some criteria used to define the topic as involving “esthetic” were: the presence of words “esthetic”, “veneer”, or “metal-free”; emphasis on the anterior teeth; tooth bleaching; techniques for restoration stratification; dental re-anatomization; orthognathic surgery (except related to temporomandibular disorders); orofacial harmonization; surgery of skeletal deformities; orthodontic aligners; gingival biotype; and digital smile design.

### Speaker`s gender classification

The speaker`s gender was also classified based on the speaker`s name. When a not gender-specific (unisex) name was found, the gender was identified by searching the speaker`s name on the internet, mainly in the curriculum registered in the Lattes platform hosted by the National Council for Scientific and Technological (CNPq/ Brazil). For lectures with more than a single speaker, the gender was classified as “both” when the male/female ratio was 1:1. Otherwise, the predominant gender was used in the classification. Finally, the distribution of male and female dentists registered in the FCD was recorded by each dental specialty. These last data were analyzed only for those specialties with more than a thousand dentists registered.

### Data analysis

Descriptive analyses of data were performed to identify the distribution of lectures according to the conference, year, dental specialty, involvement of topics related to esthetic matters, and the speaker`s gender. Chi-square tests were used to assess possible associations among the factor evaluated.

## Results

The distribution of lectures involving or not esthetic for each dental conference is presented in Table 1. The prevalence of lectures related to esthetics did not significantly change among the years evaluated. In contrast, it was observed a reduction in percentage between 2016 (35.0%) and 2018 (26.5) for the CIOBA (p = 0.046). Moreover, CIOBA (31.1%), CIOSP (34.5%), and CIOGO (36.9) showed similar mean relative numbers of lecture titles addressing topics related to esthetics during the years evaluated.

**Figure 1:**
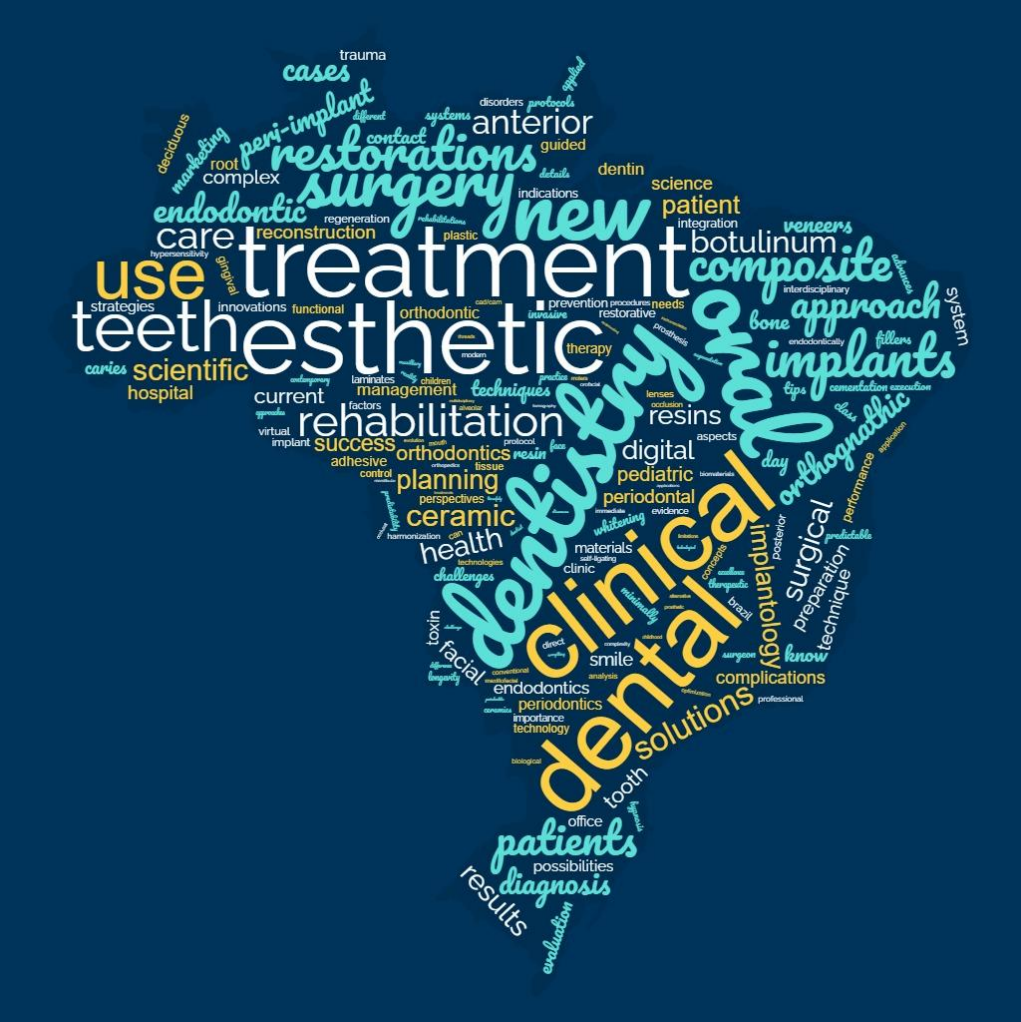
Word cloud illustrating some words found in the lecture`s titles. The font size of a word is directly proportional to its frequency in the titles.

A word cloud was generated using the words cited in the lecture’s titles, excluding prepositions and articles (Figure 1). In the figure, the font size of a word is directly proportional to its frequency in the titles. The most frequent word in the tiles was “esthetic(s)”, which was cited 212 times (13.6%), followed by “dentistry” (155; 9.9%) and “treatment” (127; 8.1%). Words referring to some oral diseases, such as “caries” (20; 1.3%), “disease” (15; 1.0%), “periodontitis” (7; 0.4%), and “Bruxism” (7; 0.4%), had relatively low frequency in the titles. On the other hand, words suggesting esthetic procedures like “resin” (86; 5.5%), “ceramic” (52; 3.3%), “botulinum toxin” (33; 2.1%), and “bleaching” (26; 1.7%) appeared more in the titles analyzed.

Table 2 summarizes the lecture titles addressing or not topics related to esthetics according to the dental specialties recognized by the FCD. As expected, no lectures dealing with esthetic matters were observed for specialties such as Community Health, Dental and Maxillofacial Radiology, Endodontics, Forensic dentistry, and others. On the other hand, the dental specialties Orofacial harmonization (96.1%), Restorative Dentistry (71.9%), and Prosthodontics (78,6%) had the highest prevalence of lecture titles describing some topics involving esthetics. A balanced distribution was observed for the specialties of Implantology (47.8%) and Periodontics (45.3%). Regardless of the topic, the highest number of lectures was observed for the specialty Restorative Dentistry (349; 22.3%), followed by Prosthodontics (289; 18.5%) and Implantology (222; 14.2%).

**Table 1:** Distribution of the number (%) of lectures according to the conference, year, and involvement or not of topic related to esthetic.

Year	2016		2017		2018		2019		2020		**Overall**	
Esthetic lecture	Yes	No	Yes	No	Yes	No	Yes	No	Yes	No	Yes	No
CIOGO	-	-	44 (37.9)	72 (62.1)	-	-	70 (35.0)	130 (65.0)	-	-	114 (36.1)	202 (63.9)
CIOBA	92* (35.0)	171 (65.0)	-	-	59* (23.6)	163 (73.4)	-	-	-	-	151 (31.1)	334 (68.9)
CIOSP	42 (29.0)	103 (71.0)	55 (39.6)	84 (60.4)	42 (32.3)	88 (67.7)	47 (32.9)	96 (67.1)	45 (40.2)	67 (59.8)	231 (34.5)	438 (65.5)
Overall	134 (32.8)	274 (67.2)	99 (38.8)	156 (61.2)	101 (28.7)	251 (71.3)	117 (34.1)	226 (65.9)	45 (40.2)	67 (59.8)	496 (33.7)	974 (66.3)

* Indicate statistical association between the year and distribution of topic related to esthetic (Chi-square test, p = 0.046).

**Table 2:** Number (%) of lectures involving or not the topic esthetic according to the dental specialties defined by the Federal Dentistry Council from Brazil.

Dental specialty	Topic involving esthetic		Overall
	Yes	No	
Community Health	0 (0.0)	34 (100.0)	34 (100.0)
Dental and Maxillofacial Radiology	0 (0.0)	32 (100.0)	32 (100.0)
Endodontics	0 (0.0)	104 (100.0)	104 (100.0)
Forensic Dentistry	0 (0.0)	18 (100.0)	18 (100.0)
Geriatric Dentistry	0 (0.0)	11 (100.0)	11 (100.0)
Implantology	106 (47.8)	116 (52.2)	222 (100.0)
Homeopathy	0 (0.0)	7 (100.0)	7 (100.0)
Maxillofacial Prosthetics	3 (75.0)	1 (25.0)	4 (100.0)
Occupational dentistry	0 (0.0)	1 (100.0)	1 (100.0)
Oral and Maxillofacial Surgery and Traumatology	31 (21.8)	111 (78.2)	142 (100.0)
Oral Pathology	0 (0.0)	27 (100.0)	27 (100.0)
Orofacial Harmonization	74 (96.1)	3 (3.9)	77 (100.0)
Orthodontics	31 (22.1)	109 (77.9)	140 (100.0)
Orthopedics	3 (9.7)	28 (90.3)	31 (100.0)
Pediatric Dentistry	9 (7.2)	116 (92.2)	125 (100.0)
Periodontics	92 (45.3)	111 (54.7)	203 (100.0)
Prosthodontics	227 (78.6)	62 (21.4)	289 (100.0)
Restorative Dentistry	251 (71.9)	98 (28.1)	349 (100.0)
Special Needs Dentistry	0 (0.0)	14 (100.0)	14 (100.0)
Sports Dentistry	0 (0.0)	18 (100.0)	18 (100.0)
Stomatology	0 (100.0)	25 (100.0)	25 (100.0)
Temporomandibular Dysfunction and Orofacial Pain	2 (5.1)	37 (94.9)	39 (100.0)
Others	42 (13.7)	264 (86.2)	306 (100.0)
Overall	862 (39.0)	1,347 (61.0)	2,209 (100.0)

The overall data does not correspond to the sum of line values since the same lecture can be classified for more than one dental specialty.

The distribution of the speaker`s gender as a function of the topic address or not esthetic matters are presented in Table 3. It was observed a statistically significant association between the speaker`s gender and the topic addressed (p < 0.001). A higher prevalence of male speakers was observed for the topics that involved esthetics.

**Table 3:** Distribution (%) of lectures involving or not the topic esthetic according to the speaker’s gender.

Topic involving esthetic	Speaker`s gender			Overall
	Male	Female	Both	
Yes	400 (81.1)	83 (16.8)	10 (2.1)	493 (100.0)
No	646 (66.7)	297 (30.6)	26 (2.7)	969 (100.0)
Overall	1,046 (71.5)	380 (26.0)	36 (2.45)	1,462 (100.0)

A significant association between the speaker`s gender and the distribution of topics related to esthetics was observed (Chi-square test, p < 0.001). The gender was not identified in eight lectures.

**Figure 2:**
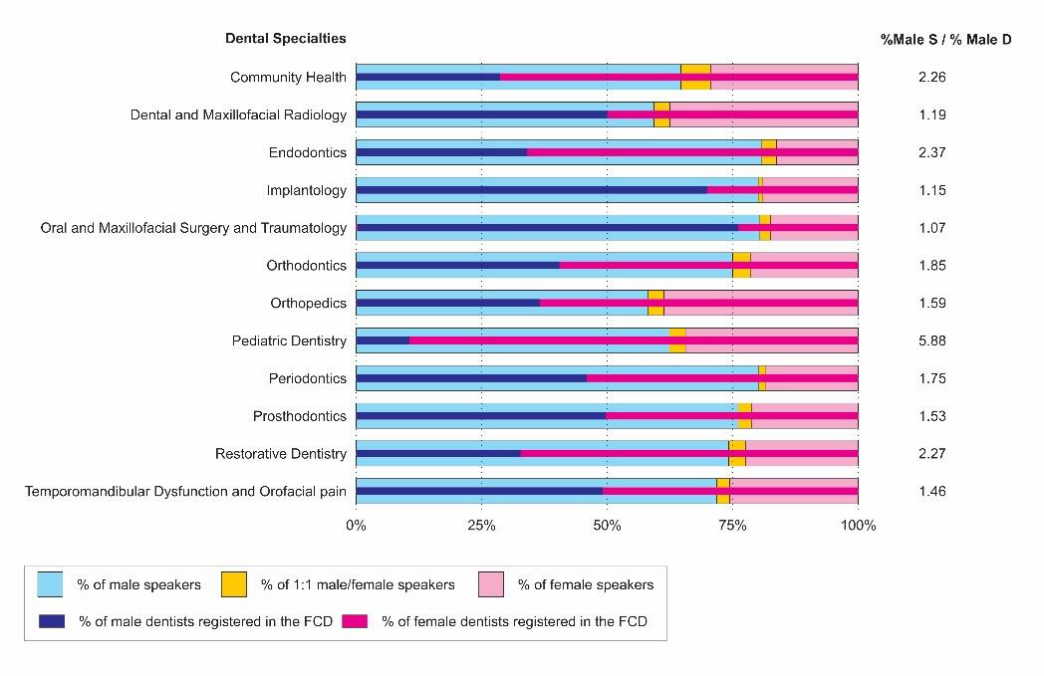
Gender percentages of speakers and dentists registered for some dental specialties in the Federal Council of Dentistry (FCD) from Brazil. % Male S = percentage of male speakers. Male D = percentage of male dentists. Note that only two dental specialties present more male dentists than females registered in the FCD. However, a predominance of male speakers was observed for all specialties.

Figure 2 illustrates the percentages of the gender of speakers (male, female, or both) and the dentits registered in the FCD according to dental specialty. Most speakers were male (overall = 73.7%) for all dental specialties. The dental specialties that presented the highest percentages of male speakers were Oral and maxillofacial surgery and traumatology (80.9%), Endodontics (80.8%), Periodontics (80.2%), and Implantology (80.1%). On the other hand, the specialties Orthopedics (61.3%), Dental and Maxillofacial Radiology (65.7%) showed the lowest prevalence of male speakers. The highest percentage of male dentists was observed for Oral and maxillofacial surgery and traumatology (75.9%), followed by Implantology (69.9%), and the lowest percentage was for Pediatric Dentistry (10.6%). Male/ female dentist rates close to one were observed for Dental and Maxillofacial Radiology (50.0:50.0%), Prosthodontics (49.6/50.4%), and Temporomandibular dysfunction and orofacial pain (49.1/50.9%). Pediatric Dentistry showed the highest discrepancy between the percentages of male speakers (62.4%) and male dentists (10.6%), resulting in a ratio of 5.88. Community health, Endodontics, and Restorative Dentistry also presented high discrepancies ranging from 2.26 to 2.37. The most balanced distribution between the genders of speakers and dentists was observed for Oral and maxillofacial surgery and traumatology (1.07), followed by Implantology (1.15). No dental specialty had a higher percentage of male dentists than those male speakers.

## Discussion

The present study's findings showed that oral diseases are barely discussed in the lectures at three of the main dental conferences in Brazil. Contrarily, esthetics was discussed in approximately one-third of the lectures in the conferences held between 2016 and 2020, even though several dental specialties do not include esthetic topics (e.g., Endodontics). For instance, dental caries in adults is strongly related to Restorative Dentistry, but more than 70% of the lectures in this specialty addressed esthetics. A possible explanation relates to clinicians seeking continuing education involving procedures that are more profitable. Therefore, dental conferences probably understand that lectures involving esthetics, with more financial return than preventing or treating some oral disorders, attract more dentists. Indeed, the artistic ability of clinicians seems to be more appraised by laypersons than the dentist`s knowledge to diagnose and treat oral diseases properly. Moreover, in the highly competitive labor market observed in Brazil, advertising esthetic procedures (e.g., in social media) tends to attract more patients. In contrast, low-income patients, who are more prone to present oral disorders, are treated in public services ^23^. Unspecialized clinicians attending oral-health public services seeking novelties for their profession may be the target of multi-professional conferences (non-specific for dentistry). Therefore, the scientific programs of large dental conferences seem to be “market-driven.”

 The dental specialties involved in oral rehabilitation or restorative procedures, which usually involve some aesthetic aspect, had the highest number of overall lectures. Restorative Dentistry (349) and Prosthodontics (289) received the highest number of lectures, and the percentage of those involving esthetics in these specialties were 71.9 and 78.6%, respectively. In contrast, only 34 lectures were classified as approaching some topic of Community Health, which is an essential specialty to control dental diseases in the Brazilian population. Similarly, a few lectures were classified for the specialties involved in significant oral diseases (e.g., oral cancer), such as Oral Pathology ^27^ and Stomatology ^25^. The number of lectures addressing these last two specialties is lower than one-tenth of those classified for Restorative Dentistry and Prosthodontics. Other important disorders treated by dentists are temporomandibular dysfunctions and orofacial pain. However, only 39 lectures were identified in the specialty responsible for diagnosing and treating these conditions. Even Orofacial Harmonization, which the FCD recognized as a dental specialty only in 2019, had almost twice more lectures as Temporomandibular Dysfunction and Orofacial Pain. It is important to emphasize that the specialists in Orofacial Harmonization should be qualified to treat pain-related conditions, but 96% of lectures associated with the specialty involved esthetic-related topics. 

Other important findings of the present study rely on the inequity in the speaker`s gender. Almost three of each four lectures were presented only by men, even though more than half (56%) of dentists registered in the FCD are women. Except for Oral and Maxillofacial Surgery and Traumatology (24.1%), and Implantology (30.1%), there are more female dentists than males for all other dental specialties. The highest prevalence (89.4%) of female dentists was observed for Pediatric Dentistry, but women gave only approximately one-third of the lectures classified in this specialty. In general, esthetic concerns are greater in women than men, including dissatisfaction with their smiles or facial features ^24^ ^,^ ^25^. Therefore, it could be expected that gender inequity would reduce for speakers involving esthetic topics. However, the prevalence of male speakers was 22% higher when the topic involved esthetics (81.6%) than otherwise (66.7%). It is important to emphasize that most speakers are dentists who work in dental schools and develop academic activities. As the academic career level increases (e.g., leadership positions), it is observed a reduction in the number of women, and this phenomenon is described as “the pipeline leaks ^26^.”

The gender inequity in speakers found in the present study agrees with other prior studies, and it is a global phenomenon ^19^ ^,^ ^20^ ^,^ ^27^. Speaking invitation entails credibility and is a professional career metric, increasing the speakers' visibility and academic work. Indeed, women are underrepresented in other academic roles. A prior study found that only one of each five North American dental schools has a woman as a dean, and less than 8% of the dental journal has a female editor-in-chief ^28^. Inequity is also observed in the authorship of articles. When the first and senior authors are analyzed, only 21% and 14%, respectively, are women ^29^. Gender inequity in the academic carrier can be attributed to several factors, including conscious and unconscious bias. In general, it has been observed that women have reduced time to dedicate to academic tasks. Career interruptions for parental leave, childcare, and unavailability to travel are some of the reasons that can help to explain gender inequity ^30^. Moreover, structural misogyny remains in several societies, and even women without children are less prone to achieve the highest positions in an academic career ^21^ ^,^ ^22^.

 The present study showed that esthetic topics tend to be more prevalent in Brazilian dental conferences than those associated with oral disorders. Considering that several oral conditions remain as burden diseases, the definition of lecture contents in dental conferences should be driven to entitle the dentist to solve more common diseases affecting the patient’s quality of life. However, it is important to be aware that this is a challenging change since esthetic procedures usually enhance dental offices' profitability. This point is even more important in a competitive labor market, as observed in Brazilian dentistry. Besides, reducing gender inequity in the speakers is another challenge. Brazil has one of the lowest gender inequities when the authorship of publications in dental journals is evaluated ^21^, indicating a high number of well-qualified females involved with dentistry in the country. Then, a solution for the gender gap in conferences could rely on simply developing policies to increase the number of female speakers. In addition to actions related to the speaker`s gender, improving the representativity (e.g., more black people) among the speakers could favor society's development. An important limitation of the present study was that the classifications were based only on the lecture`s title and speaker`s name. The lectures' contents were unavailable, and it is possible some misclassifications. Moreover, although only three dental conferences were analyzed in the present study, these represent three different Brazilian geographic regions. The number of conferences analyzed was limited because to reduce the risk of bias during the qualitative screening of topics and speakers associated with each lecture. Besides, it is unlike that the study's main findings would be modified. Further to the three selected, the International Dental Conference of Rio de Janeiro (CIORJ) is among the largest conferences in the country. However, this last was not set due to be in the same geographic region as CIOSP. Future studies could evaluate the lecture`s topic and speakers’ gender in smaller conferences, such as those organized by dental schools. Another interesting question worth investigating is the possible differences between sponsored lectures and those funded only by the conference budget.

In conclusion, we observed a high prevalence of lectures involving esthetic topics in three major dental conferences held in Brazil between 2016 and 2018, with no significant changes during the period evaluated. Besides, an important gender gap was observed among the speakers, with a high prevalence of men in all dental specialties. The gender discrepancy was higher in lectures involving esthetics.
